# Youth sexual offending in Hong Kong: examining the role of self-control, risky sexual behaviors, and paraphilic interests

**DOI:** 10.3389/fpsyt.2023.1143271

**Published:** 2023-06-12

**Authors:** Heng Choon (Oliver) Chan

**Affiliations:** Department of Social Policy, Sociology, and Criminology, University of Birmingham, Birmingham, United Kingdom

**Keywords:** sexual offending, youth, self-control, risky sexual behavior, paraphilic interest, Hong Kong

## Abstract

**Introduction:**

Little is known about the nature and prevalence of sexual offending among youth in Hong Kong.

**Methods:**

Testing self-control theory and sexual health risk factors (i.e., risky sexual behaviors [general and two subtypes] and paraphilic interests [general and 14 subtypes]), the prevalence of self-reported sexual offending behaviors (i.e., threat of sexual assault, penetrative sexual assault, and nonpenetrative sexual offense) was examined in a community-based sample of 863 young people (aged 17 to 20) in Hong Kong.

**Results:**

In this study, men reported significantly higher levels of threat of sexual assault and of general and 12 subtypes of paraphilic interests than women; and women reported a significantly higher level of a specific paraphilic interest subtype (i.e., transvestic fetishism) than men. Logistic regressions found that, in general, a low level of self-control and high levels of risky sexual behaviors and paraphilic interests were important factors in the participants’ likelihood of issuing threats of sexual assault and engaging in penetrative and nonpenetrative sexual assault.

**Discussion:**

Important practical implications for reducing the tendency of young people to engage in sexual offending behavior can be derived from this study.

## Introduction

Unlike studies conducted in the West, most sexual offending studies conducted in the East Asian region involved intimate partners as the offenders (sexual intimate partner violence [IPV]) (1). Little is known about those general sex offenses committed by strangers or acquaintances. In Hong Kong, the lifetime prevalence rate of general sexual victimization, such as sexual IPV and child sexual abuse (CSA), is ranged from below 1 to 16% (2–6). For example, Chan (2) found that 16% of his sample of 1,171 adults (aged 18–40) (11% men and 19% women) reported being a victim of sexual offending, of which 3% (1% men and 5% women) and 19% (15% men and 22% women) were penetrative and nonpenetrative sexual victimization, respectively. Concerning the sexual offending perpetration, 12% of adults (16% men and 9% women) reported to have committed a sexual offense at least once in their life [2% penetrative (2% men and women) and 6% nonpenetrative (8% men and 5% women)]. Five percents of men and women involved in both general sexual offending perpetration and victimization (i.e., the victim-offender overlap).

In Chan et al. (7) study of 1,154 Chinese Hong Konger adults who engaged in dating relationships, the unwanted touch (65%) was the most frequently reported sexual abusive acts of those who had experienced CSA. The sexual IPV was estimated at 9% lifetime and 5% past-year prevalence rate. Another study by Chan et al. (7) of 5,049 Chinese adults reported the CSA and ASV lifetime prevalence rates to be 0.9% (0.7% unwanted touch and 0.2% forced sex) and 0.8% (0.4% unwanted touch, 0.2% forced sex, and 0.2% sexual coercion), respectively. The prevalence rate of CSA was much higher in women (1.1%) than men (0.6%), while men (0.8%) engaged more ASV against nonpartners than women (0.6%).

Prevalence studies have generally suggested that a quarter of all sexual offenses are perpetrated by juvenile offenders (8). In the U.S., juvenile sexual offenders are consistently accounted for 15 to 20% of all sexual offenses (9). According to Barbaree et al. (10), offenders under 18 are accountable for 20% of all rapes, half of all CSA cases, and one-third of all sexual offenses against other adolescents. Most sex offenders are men and many began sexual offending in adolescence (11). In Hong Kong, the estimates of police arrest in rape have varied in recent years, with 121 police arrests in 2012 to 65 police arrests in 2017, and lastly 79 arrests in 2021 (12). However, there is a downward trend in arrests for indecent assaults (e.g., sexual molestation) was observed (1,495 arrests in 2012, 1,077 arrests in 2017, and 1,018 arrests in 2021). According to Hong Kong Police Force (12), the juvenile (i.e., aged 10–15) and youth (i.e., aged 16–20) crime rates increased steeply in the late 20th century, which peaked in the late 1980s and early 1990s. Over the last 10 years, an initial sharp decline was documented in the arrest of juveniles and youths, but it followed by a steady increase afterwards (12).

Understanding youth sexual offending remains pertinent from the perspectives of criminal justice and public health. Over the years, studies that examined sexual offending risk factors have mainly sampled adult offenders (13), with limited information available about risk factors among juvenile sex offenders [e.g., (14–17)]. Given the scarcity of research conducted in Hong Kong on youth sexual offending, the present study is important to investigate not only the prevalence of self-reported sexual offending behaviors but also the psychosocial and sexual health risk factors of sexual offending behaviors among Hong Kong youth. Importantly, this study fills a gap in the literature by drawing from the rarely examined population of Hong Kong youth. It remains uncertain if the findings found in research commonly conducted with Western samples (e.g., in Canada and the U.S.) can be similarly applied to other countries, and especially in the Asian populations. This information can be informative for behavioral scientists and mental health professionals to design and conduct offender rehabilitation strategies and crime preventive measures. Timely and effective interventions are key to prevent offenses and potential escalation in offending severity.

### Psychosocial and sexual health risk factors

The self-control theory asserts that those with a lower self-control are more inclined to engage in delinquent and criminal behavior in return for an immediate gratification, without considering the possible consequences of their action (18). There are six primary psychosocial characteristics of those who are low in self-control: They are impulsive, short-tempered, self-centered, risk-seeking, and desire physical activities (over mental activities) and simple tasks (over complex tasks) (2, 19). Vazsonyi and Klanksek (20) postulated that this personality trait is relatively stable over the lifespan and across individuals irrespective of their demographics, including sex, age, culture, and social class. Recent studies in criminology demonstrate that men possess significantly lower self-control than women [e.g., (21, 22)], while Ridder et al. (23) meta-analysis of 102 studies observed that the positive relationship between trait self-control and deviant behaviors was also stronger in men than in women.

Recent empirical studies in sexual offending consistently reported that low self-control is a significant predictor of deviant and criminal sexual interests and behaviors (24, 25). Sex offenders with low self-control are more likely to be impulsive, aggressive, physical, risk-seeking, insensitive, and short-sighted. Nonetheless, DeLisi and Wright (26) asserted that a curvilinear relationship can be observed between low self-control and sexual murder. Compared to sexual killers who are high in self-control, those with low self-control may be more disorganized in their offending pattern.

In contrast, risky sexual behaviors and paraphilic interests can be regarded as sexual health risk factors. Risky sexual behaviors (e.g., unprotected sexual intercourse and multiple sexual partners) can lead to significant health problems, including outcomes of enduring poor reproductive health (e.g., unintended pregnancy, infertility, and sexually transmitted infections [STIs]) (27, 28). Compared with older adults, adolescents and young adults are in a higher tendency to involve in risky sexual behaviors as sexual exploration typically begins in adolescence and young adulthood (29). Significant neurobiological changes in adolescence such as an increase in urges for impulsive behavior and reward seeking (30, 31) can result in the learning of sexual encounters that are impulsive through expectancy formation (32). Moreover, the effect can be further exacerbated as adolescence often involved increased peer influences on behavior and reduced parental monitoring (33, 34). As a result, global studies (e.g., in Addis Ababa, Thailand, and the US) have constantly indicated that adolescents and young adults are in a higher likelihood to have adverse sexual health outcomes as a result of their higher tendency of unprotected sexual intercourse with multiple partners (35–38).

Based on the developmental and life-course approach (39) and criminal career perspective (40), it is plausible that risky sexual behavior can be escalated to sexual offending (e.g., sexual assault, rape). Moreover, this notion is similar with the confluence model of sexual aggression, in which those who prefer impersonal sex impersonal sex (e.g., engaging in casual sexual relationships, and having multiple sexual partners concurrently or in the past), coupled with hostile masculinity traits (e.g., misogynistic attitudes), are the most likely to engage in sexual violence (41). As such, impersonal sex can be regarded as risky sexual behavior. In their review of global studies, Davis et al. (42) found a positive relationship between men’s type of sexual partner (e.g., more lifetime sexual partners, engaging in concurrent or extramarital sex, having sex with a high-risk partner [e.g., someone who uses intravenous drugs], engaging in transactional sex [e.g., hire a prostitute] with women for sex), condom use (e.g., nonconsensual sex without a condom and inconsistent condom use), and history of STI diagnosis or symptoms and their sexual violence perpetration. There have been mixed findings on sex differences in adolescents’ risky sexual behaviors. Although most studies have reported that men engage in significantly more risky sexual behaviors than women [e.g., (21, 43, 44)], some studies have either reported that women are sexually riskier than men (45) or found no significant sex difference (46).

Broadly speaking, paraphilias are psychological traits characterized by persistent unconventional sexual interests. According to the American Psychiatric Association (47), paraphilias is an “intense and persistent sexual interest other than sexual interest in genital stimulation or preparatory fondling with phenotypically normal, physically mature, consenting human partners” (p. 685). However, paraphilic (or atypical) interest is described as sexual arousal from an atypical sexual activity (e.g., exposing one’s genitals to nonconsenting others) or target (e.g., prepubescent children) (48). Yet, it may not necessarily be pathological if someone is having a paraphilic interest or acting on one. Furthermore, paraphilia can only be clinically diagnosed if the paraphilic interest is recurrent, persistent, essential for sexual enjoyment, and leads to substantial distress or weakened occupational functioning. Recent studies [e.g., (49–51)] found that men generally are less repulsive (or more sexual arousal) than women for most paraphilic interests [e.g., (49–51)]. Of note, these studies recruited mostly nonclinical samples.

Nonetheless, paraphilic interests have been positively associated with subsequent involvement in sexual activities that are paraphilic in nature (52–54). These activities can include sexual offending behavior if acted out upon nonconsenting individuals (e.g., sadism, biastophilia, and pedophilia). Hence, paraphilic interests (e.g., biastophilia, pedophilia, and sadism) can be regarded, to a large extent, a contributing factor in some sexual offenses (55). Indeed, Drury et al. (56) found in their study that most sexual offender inmates were diagnosed with at least one paraphilia (57% pedophilia, 26% exhibitionism, and 21% voyeurism) and they tended to suffer from traumatic childhood experiences (e.g., paternal abandonment or neglect, and different types of child abuse). However, it should also be noted that not all diagnosed with paraphilia act upon their sexual interests and many sexual offenders are not paraphilic (57, 58).

In the conventional society, sexual practices are commonly related to the society’s kinship structures and power gradients, which typically adhere to the prescribed and shared cultural scripts that encourage or discourage certain types of sexual interests and behavior (59). Compare to Western cultures, Asian and Middle Eastern cultures generally adopt a more restrictive view of sexual issues. Sex has always been and remains a taboo subject of discussion in these cultures (60, 61).

## The present study

Hong Kong has been a semi-autonomous city (Special Administrative Region) of the People’s Republic of China (PRC) since July 1, 1997. Hong Kong was a British colony for over 150 years. Inhabiting a modern Chinese society that is also a major regional financial hub, Hong Kongers (about 95% of whom are of Chinese descent) commonly balance a modern Western lifestyle with traditional Chinese cultural values and practices.

This exploratory study has two primary aims. First, it explored self-reported sexual offending behaviors (i.e., threat of sexual assault, penetrative sexual assault, and nonpenetrative sexual offense) within an under-researched population, namely Hong Kong youth. Second, it can be regarded a pioneer study to investigate the role of psychosocial (i.e., self-control) and sexual health (i.e., risky sexual behaviors and paraphilic interests) correlates in sexual offending perpetration in a Hong Kong community-based sample. Specifically, it remains unclear whether these psychosocial and sexual health risk factors are applicable to describe sexual offending behavior in a Chinese cultural context.^1^ The study findings have potential implications for practice (e.g., crime prevention) by identifying significant psychosocial and sexual health predictors for different kinds of sexual offending behaviors. Strategic and timely interventions that focus on these risk factors could help to reduce young people’s propensity to engage in sexual offending behaviors. There are two research hypotheses set forth in this study.

*Hypothesis 1*: There are sex differences in the mean levels of different types of psychosocial (i.e., self-control) and sexual health (i.e., general and two subtypes of risky sexual behaviors,^2^ and general and 14 subtypes of paraphilic interests^3^) risk factors, such that young men are expected to have a lower mean level of self-control and higher mean levels of risky sexual behaviors and paraphilic interests than young women.

*Hypothesis 2*: Psychosocial and sexual health risk factors are associated with different types of sexual offending behaviors (i.e., threat of sexual assault, penetrative sexual assault, and nonpenetrative sexual offense) in youth, even when controlling for demographic characteristics (i.e., sex, religiosity, and intimate relationship status), such that a low level of self-control and high levels of risky sexual behaviors and paraphilic interests are associated with all types of sexual offending behaviors.

## Methods

### Participants and procedure

In this study, 863 participants aged 17 to 20 were recruited from eight public (i.e., government-funded) and three private universities in Hong Kong.^4^ Most students who enter universities immediately after completing their secondary education are 17 or 18 years of age. The participants were either recruited within university compounds randomly (about 55%; e.g., student cafeterias, libraries, and common areas) or through a convenience sampling approach (about 45%; e.g., recruitment from classrooms with prior consent from the instructors). The participants’ informed consent was acquired prior to the administration of the survey. The participants were offered the option of completing either a printed (about 20%) or online (i.e., Qualtrics Survey; about 80%) questionnaire. Confidentiality was promised and their anonymous responses would only be used for research purposes. This was a voluntary participation without any monetary incentive. The participants averagely used 25 min to complete the questionnaire.

In this study, 66.9% were women and 33.1% were men. The mean age of the full sample was 19.11 (*SD* = 0.89, range 17–20).^5^ Men and women had a mean age of 19.09 years (SD = 0.94) and 19.12 years (*SD* = 0.86), respectively; this difference was not significant. Most of the participants (86.1%) were Hong Kong locals, nearly three quarters (71.3%) were single, more than one third (36.7%) had obtained post-secondary education, and about three quarters (70.6%) reported having no religious beliefs.

### Measures

In this study, English and Chinese versions of the self-reported questionnaire were used to accommodate the different language abilities of the participants. To accommodate the local Chinese population, the English-language scales were first translated by an experienced and academically qualified English-to-Chinese translator. The Chinese versions were subsequently translated back into English to ensure face validity, and the back-translations were compared with the original English scales to determine their content similarity. A pilot study (with 20 participants) was conducted, which resulted in several items translated into Chinese were revised after the pilot.

#### Sexual offending behaviors

To assess the participants’ lifetime experience of sexual offending perpetration behavior, three questions were asked on whether they had (a) issued a threat of sexual assault, (b) engaged in penetrative sexual assault, and/or (c) engaged in nonpenetrative sexual offense. This measure was dichotomized (0 = *no*, 1 = *yes*). If the participants admitted to have engaged in sexual offending behavior, they were then asked about the type of sexual behavior they performed (i.e., penetrative or nonpenetrative). Sample items asked whether the participants had “Threatened to use force or harmed her/him to have sexual contact against her/his will” and “Exploited the fact that she/he was unable to resist (e.g., after she/he had too much alcohol or another drug) to have sexual contact or intercourse against her/his will.” Cronbach’s α for this measure was 0.86 (men = 0.84, women = 0.88).

#### Self-control

To measure the participants’ levels of self-control, the widely used 23-item Low Self-Control Scale was adopted (64) to operationalize the six core elements of self-control (i.e., impulsivity, self-centeredness, risk-seeking, volatile temper, preference for simple tasks, and preference for physical activities). This scale was measured on a 4-point Likert scale (1 = *strongly agree*, 4 = *strongly disagree*) for a total score ranging from 23 to 92, with a higher score indicating greater self-control. Sample items include “Sometimes I will take a risk just for the fun of it” and “I often act in the spur of the moment without stopping to think.” Cronbach’s α for this measure was 0.86 (men = 0.86, women = 0.86).

#### Risky sexual behaviors

To measure the participants’ levels of involvement in risky sexual behaviors over the past 6 months, the 23-item Sexual Risk Survey (65) was used. This measure was dichotomized (0 = *no*, 1 = *yes*) and had two subscales (i.e., 16 items on penetrative and 7 items on nonpenetrative risky sexual behavior). The total score ranged from 0 to 23, with a higher score denoting a greater involvement in risky sexual behaviors. Sample items include “Had anal sex without a condom,” (penetrative risky sexual behavior) and “Had left a social event with someone you just met” (nonpenetrative risky sexual behavior). Cronbach’s α for this measure was 0.90 (men = 0.92, women = 0.88).

#### Paraphilic interests

To measure the participants’ interest in paraphilic activities, a 40-item Paraphilia Scale was used (66). This measure was assessed on a 7-point Likert scale (−3 = *very repulsive*, +3 = *very arousing*), with a total score ranging from −120 to +120. Of the 40 items, 32 were constructed to measure 14 subtypes of paraphilic interests.^6^ A higher score denoted greater interest in the corresponding paraphilic activities. Sample items include “You are kissing, fondling, and touching someone’s feet” (fetishism) and “You are spanking, beating, or whipping someone” (sadism). Notably, two items in this measure were regarded as control items in the original study, as they referred to interest in adult men and women (66). Cronbach’s α for this measure was 0.99 (men = 0.98, women = 0.99).

### Data analysis plan

Independent samples *t*-tests were used to explore the sex differences in self-control and different types of risky sexual behaviors (i.e., general, penetrative, and nonpenetrative risky sexual behavior), while Mann–Whitney *U* tests were performed on the general and 14 subtypes of paraphilic interests as the distribution of these constructs was highly skewed. Binary logistic regressions were then computed to investigate the effects of different psychosocial and sexual health risk factors on different types of sexual offending behaviors (i.e., threat of, penetrative, and nonpenetrative sexual assault) while controlling for the participants’ demographic characteristics (i.e., sex, religiosity, and intimate relationship status). Two logistic regression models were tested on each type of sexual offending behavior with Model I to demonstrate the overall effect of general risky sexual behaviors and general paraphilic interests, while Model II to identify the individual effect of different types of risky sexual behaviors and paraphilic interests (67). These demographic characteristics were expected to be potential confounders in the model. The participants’ religiosity was assessed by how religious they perceived themselves to be on a 6-point Likert scale (1 = *not at all*, 6 = *very strongly*). The Pearson correlations of the tested constructs were calculated. No correlation at or above 0.70 was found, indicating no collinearity.

## Results

### Lifetime prevalence of sexual offending behavior

Of the full sample, 17.7% reported that they had issued a threat of sexual assault at least once in their lifetime (see Table 1). A significant sex difference was observed (*p* = 0.014). Besides, 7.3% (8.4% d 6.8% women) and 14% (16.1% men and 13% women) of the participants reported engaging in penetrative and nonpenetrative sexual offending behaviors, respectively. No significant sex differences were found.

**Table 1:** Prevalence and sex differences of sexual offending behavior (*N* = 863).

	All sample		Men		Women			
Variable	(*N* = 863)		(*n* = 286)		(*n* = 577)		Sex differences	
	*N*	Percent	*n*	Percent	*n*	Percent	*χ* ^2^	Phi
Threat of sexual assault	153	17.7%	64	22.4%	89	15.4%	6.34	0.09 *
Penetrative sexual assault	63	7.3%	24	8.4%	39	6.8%	0.75	0.03
Nonpenetrative sexual offense	121	14.0%	46	16.1%	75	13.0%	1.51	0.04

### Mean differences of psychosocial and sexual health risk factors

In Table 2, significant sex differences in the participants’ paraphilic interests were observed. Relative to women, men reported significantly higher levels of (were more favorable about) general paraphilic interests (*U* = 70373.50, *p* < 0.001), voyeurism (*U* = 62061.00, *p* < 0.001), exhibitionism (*U* = 70311.50, *p* < 0.001), scatologia (*U* = 71843.00, *p* = 0.001), fetishism (*U* = 71525.00, *p* = 0.001), frotteurism (*U* = 67744.00, *p* < 0.001), sadism (*U* = 71915.00, *p* = 0.002), biastophilia (*U* = 66182.00, *p* < 0.001), urophilia (*U* = 72637.50, *p* = 0.002), scatophilia (*U* = 74628.50, *p* = 0.010), hebephilia (*U* = 71385.50, *p* = 0.001), pedophilia (*U* = 69723.00, *p* < 0.001), and zoophilia (*U* = 75023.50, *p* = 0.008). However, women reported higher levels of transvestic fetishism (*U* = 75822.00, *p* = 0.048) than men. No significant sex differences were found in levels of self-control and risky sexual behaviors.

**Table 2:** Mean level differences of psychosocial and sexual health risk factors.

	All sample		Men		Women		
Risk factors	(*N* = 863)		(*n* = 286)		(*n* = 577)		
	M	SD	M	SD	M	SD	*t*/*Z*
			/Mean rank		/Mean rank		value
Self-control	59.34	9.42	58.52	9.58	59.74	9.32	−1.79
*Risky sexual behaviors*							
General behaviors	1.28	2.85	1.43	3.27	1.21	2.61	1.02
Penetrative behaviors	0.70	1.95	0.79	2.31	0.66	1.74	0.95
Nonpenetrative behaviors	0.58	1.19	0.64	1.27	0.55	1.14	1.09
*Paraphilic interests*							
General interests	−58.73	54.05	474.44		410.96		−3.52 ***
Voyeurism	−1.42	1.80	502.50		396.24		−6.29 ***
Exhibitionism	−1.84	1.62	472.29		410.57		−3.86 ***
Scatologia	−1.57	1.70	469.30		413.51		−3.31 **
Fetishism	−2.53	4.19	470.41		412.96		−3.20 **
Tranvestic fetishism	−2.35	3.06	408.61		443.59		−1.97 *
Frotteurism	−1.46	1.77	482.63		406.11		−4.54 ***
Sadism	−8.14	8.88	469.05		413.64		−3.09 **
Masochism	−8.66	8.98	452.53		421.83		−1.72
Biastophilia	−3.28	3.29	488.09		403.40		−4.99 ***
Urophilia	−3.86	3.02	466.52		414.89		−3.16 **
Scatophilia	−4.09	2.98	458.56		418.06		−2.58 **
Hebephilia	−3.54	3.03	470.90		412.72		−3.39 **
Pedophilia	−3.89	3.06	475.71		409.55		−4.09 ***
Zoophilia	−2.05	1.55	458.18		419.02		−2.65 **

*p < 0.05, **p < 0.01, ***p < 0.001.

### Effects of psychosocial and sexual health risk factors on sexual offending behavior

Binary logistic regressions were performed to investigate the effects of psychosocial and sexual health risk factors on the participants’ self-reported sexual offending behaviors (i.e., threat of sexual assault, penetrative sexual assault, and nonpenetrative sexual offense), while controlling for their demographic characteristics (i.e., sex, religiosity [a 6-point Likert scale measure], and intimate relationship status). The results shown in Table 3 indicate that all of the regression models were significant (Model I: general sexual offending behaviors and paraphilic interests, Model II: subtypes of sexual offending behaviors and paraphilic interests). Self-control (Models I and II: *B* = −0.06, *SE* = 0.01, *p* < 0.001), general risky sexual behaviors (Model I: *B* = 0.09, *SE* = 0.03, *p* = 0.001), penetrative risky sexual behaviors (Model II: *B* = 0.11, *SE* = 0.06, *p* = 0.049), and paraphilic interests in urophilia (Model II: *B* = −0.21, *SE* = 0.10, *p* = 0.032) and zoophilia (Model II: *B* = 0.25, *SE* = 0.14, *p* = 0.049) were significantly associated with the participants’ likelihood of reporting having issued a threat of sexual assault. Being a man (Model I: *B* = 0.54, *SE* = 0.20, *p* = 0.006; Model II: *B* = 0.55, *SE* = 0.22, *p* = 0.011) and non-single (Model I: *B* = −0.60, *SE* = 0.20, *p* = 0.003; Model II: *B* = −0.64, *SE* = 0.21, *p* = 0.002) were also found to be significantly associated with having issued a threat of sexual assault.

**Table 3:** Binary logistic regression models of self-reported sexual offending behavior.

	Threat of sexual assault			
Predictors	Model I		Model II	
Demographic characteristics	*b* (*SE*)	OR (CI)	*b* (*SE*)	OR (CI)
Sex (0 = woman, 1 = man)	0.54 (0.20)	1.71 (1.17, 2.52)	0.55 (0.22)**	1.74 (1.14, 2.65)*
Religiosity	0.01 (0.06)	1.01 (0.89, 1.15)	−0.01 (0.07)	0.99 (0.87, 1.13)
Intimate relationship status (0 = nonsingle, 1 = single)	−0.60 (0.20)	0.55 (0.37, 0.82) **	−0.64 (0.21)	0.53 (0.35, 0.79) **
Psychosocial risk factor				
Self-control	−0.06 (0.01)	0.94 (0.93, 0.96)***	−0.06 (0.01)	0.94 (0.92, 0.96) ***
Sexual health risk factors				
Risky sexual behaviors	0.09 (0.03)	1.10 (0.99, 1.00)**		
Penetrative behavior			0.11 (0.06)	1.11 (0.99, 1.24) *
Nonpenetrative behavior			0.11 (0.09)	1.11 (0.93, 1.33)
Paraphilic interests	−0.01 (0.01)	0.99 (0.99, 1.00)^+^		
Voyeurism			−0.02 (0.11)	0.98 (0.80, 1.22)
Exhibitionism			0.01 (0.13)	1.01 (0.78, 1.31)
Scatologia			0.09 (0.11)	1.09 (0.89, 1.34)
Fetishism			0.03 (0.03)	1.03 (0.96, 1.10)
Tranvestic fetishism			0.01 (0.05)	1.00 (0.90, 1.11)
Frotteurism			−0.05 (0.10)	0.95 (0.78, 1.17)
Sadism			−0.02 (0.03)	0.98 (0.92, 1.05)
Masochism			−0.03 (0.03)	0.97 (0.92, 1.03)
Biastophilia			−0.03 (0.08)	0.97 (0.83, 1.13)
Urophilia			−0.21 (0.10)	0.82 (0.67, 0.98) *
Scatophilia			0.01 (0.10)	1.00 (0.83, 1.20)
Hebephilia			0.12 (0.08)	1.13 (0.96, 1.34)
Pedophilia			0.01 (0.11)	1.01 (0.82, 1.24)
Zoophilia			0.25 (0.14)	1.29 (0.98, 1.70) *
Constant	1.60 (0.63)	4.97 **	1.83 (0.67)	6.23 **
Model *χ*^2^	35.08 ***		51.25 ***	
Nagelkerke *R*^2^	0.13		0.16	
Hosmer-Lemeshow test	4.47		13.53	

	Penetrative sexual assault			
Predictors	Model I		Model II	
Demographic characteristics	*b* (*SE*)	OR (CI)	*b* (*SE*)	OR (CI)
Sex (0 = woman, 1 = man)	0.24 (0.29)	1.27 (0.72, 2.23)	0.22 (0.32)	1.24 (0.66, 2.32)
Religiosity	−0.02 (0.09)	0.98 (0.82, 1.18)	0.01 (0.10)	1.00 (0.83, 1.22)
Intimate relationship status (0 = nonsingle, 1 = single)	−0.53 (0.28)	0.59 (0.34, 1.02) *	−0.58 (0.30)	0.56 (0.31, 1.00) *
Psychosocial risk factor				
Self-control	−0.04 (0.01)	0.96 (0.94, 0.99) **	−0.05 (0.01)	0.96 (0.93, 0.98) **
Sexual health risk factors				
Risky sexual behaviors	0.17 (0.03)	1.19 (1.11, 1.27) ***		
Penetrative behavior			0.24 (0.08)	1.28 (1.10, 1.48) **
Nonpenetrative behavior			0.08 (0.13)	1.08 (0.84, 1.39)
Paraphilic interests	−0.01 (0.01)	0.99 (0.99, 1.00)*		
Voyeurism			0.12 (0.16)	1.13 (0.82, 1.55)
Exhibitionism			0.17 (0.21)	1.18 (0.79, 1.77)
Scatologia			0.08 (0.15)	1.09 (0.80, 1.47)
Fetishism			0.04 (0.05)	1.04 (0.95, 1.14)
Tranvestic fetishism			0.01 (0.08)	1.01 (0.87, 1.17)
Frotteurism	−0.39 (0.17)	0.68 (0.48, 0.95) *		
Sadism			0.04 (0.05)	1.04 (0.95, 1.14)
Masochism			−0.06 (0.04)	0.94 (0.86, 1.02)
Biastophilia			−0.06 (0.12)	0.95 (0.75, 1.19)
Urophilia			−0.32 (0.16)	0.73 (0.54, 0.98) *
Scatophilia			0.18 (0.14)	1.19 (0.90, 1.58)
Hebephilia			−0.09 (0.13)	0.91 (0.70, 1.18)
Pedophilia			−0.03 (0.16)	0.97 (0.70, 1.34)
Zoophilia			0.51 (0.20)	1.67 (1.13, 2.48) *
Constant	0.64 (0.89)	1.53 *	0.25 (0.94)	1.78 *
Model *χ*^2^	37.52 ***		54.20 ***	
Nagelkerke *R*^2^	0.13		0.18	
Hosmer-Lemeshow test	8.29		16.78	

	Nonpenetrative sexual offense			
Predictors	Model I		Model II	
Demographic characteristics	*b* (*SE*)	OR (CI)	*b* (*SE*)	OR (CI)
Sex (0 = woman, 1 = man)	0.31 (0.21)	1.37 (0.90, 2.09)	0.33 (0.24)	1.39 (0.87, 2.21)
Religiosity	0.05 (0.07)	1.05 (0.91, 1.20)	0.01 (0.07)	1.01 (0.88, 1.16)
Intimate relationship status (0 = nonsingle, 1 = single)	−0.65 (0.22)	0.52 (0.34, 0.80) **	−0.72 (0.23)	0.49 (0.31, 0.76) **
Psychosocial risk factor				
Self-control	−0.05 (0.01)	0.95 (0.93, 0.97) ***	−0.05 (0.01)	0.95 (0.93, 0.97) ***
Sexual health risk factors				
Risky sexual behaviors	0.07 (0.03)	1.07 (1.01, 1.14) *		
Penetrative behavior			0.05 (0.06)	1.05 (0.93, 1.18)
Nonpenetrative behavior			0.20 (0.10)	1.22 (1.01, 1.47) *
Paraphilic interests	−0.01 (0.01)	0.99 (0.99, 1.00)^+^		
Voyeurism			−0.02 (0.12)	0.98 (0.77, 1.24)
Exhibitionism			0.13 (0.15)	1.14 (0.86, 1.52)
Scatologia			0.08 (0.12)	1.08 (0.86, 1.36)
Fetishism			0.05 (0.04)	1.06 (0.98, 1.14)
Tranvestic fetishism			−0.02 (0.06)	0.98 (0.87, 1.10)
Frotteurism	0.02 (0.11)	1.02 (0.82, 1.26)		
Sadism			−0.01 (0.04)	1.00 (0.93, 1.08)
Masochism			−0.04 (0.03)	0.96 (0.89, 1.02)
Biastophilia			−0.21 (0.09)	0.81 (0.68, 0.97)*
Urophilia			−0.19 (0.11)	0.83 (0.67, 1.02)+
Scatophilia			0.02 (0.10)	1.02 (0.83, 1.25)
Hebephilia			0.16 (0.09)	1.17 (0.98, 1.40)+
Pedophilia			0.04 (0.12)	1.04 (0.82, 1.31)
Zoophilia			0.27 (0.15)	1.31 (0.97, 1.76)*
Constant	1.22 (0.67)	3.40*	1.35 (0.72)	3.88*
Model *χ*^2^	20.64**		50.77***	
Nagelkerke *R*^2^	0.10		0.15	
Hosmer-Lemeshow test	14.01		11.36	

Unstandardized beta (B) and standard error (SE).

+ *p* < 0.10.

*p < 0.05, **p < 0.01, ***p < 0.001.

In terms of penetrative sexual assault, low levels of self-control (Model I: *B* = −0.04, *SE* = 0.01, *p* = 0.004; Model II: *B* = −0.05, *SE* = 0.01, *p* = 0.001), general paraphilic interests (Model I: *B* = −0.01, *SE* = 0.01, *p* = 0.049), and specific paraphilic interests in frotteurism (Model II: *B* = −0.39, *SE* = 0.17, *p* = 0.024) and urophilia (Model II: *B* = −0.32, *SE* = 0.16, *p* = 0.039) were significantly associated with the participants’ propensity to engage in penetrative sexual assault. Furthermore, increased levels of general sexual risky behaviors (Model I: *B* = 0.17, *SE* = 0.03, *p* < 0.001) and penetrative sexual risky behaviors (Model II: *B* = 0.24, *SE* = 0.08, *p* = 0.001) and paraphilic interest in zoophilia (Model II: *B* = 0.51, *SE* = 0.20, *p* = 0.011) were significant predictors of penetrative sexual assault. Being non-single (Model I: *B* = −0.53, *SE* = 0.28, *p* = 0.049; Model II: *B* = −0.58, *SE* = 0.30, *p* = 0.049) was also found to be significantly correlated with the tendency to engage in penetrative sexual assault.

Significant risk factors were observed for nonpenetrative sexual offense. The participants’ levels of self-control (Models I and II: *B* = −0.05, *SE* = 0.01, *p* < 0.004) and paraphilic interest in biastophilia (Model II: *B* = −0.21, *SE* = 0.09, *p* = 0.024) were negatively correlated, whereas general risky sexual behaviors (Model I: *B* = 0.07, *SE* = 0.03, *p* = 0.023), nonpenetrative risky sexual behaviors (Model II: *B* = 0.20, *SE* = 0.10, *p* = 0.043), and paraphilic interest in zoophilia (Model II: *B* = 0.27, *SE* = 0.15, *p* = 0.049) were positively related to the likelihood of having perpetrated nonpenetrative sexual offense. Also, being non-single (Model I: *B* = −0.65, *SE* = 0.22, *p* = 0.003; Model II: *B* = −0.72, *SE* = 0.23, *p* = 0.001) was significantly associated with the participants’ tendency to commit nonpenetrative sexual offense.

## Discussion

In this sample of Hong Kong youth, the lifetime prevalence of the self-reported sexual behaviors in this study was 17.7% for issuing a threat of sexual assault, 7.3% for engaging in penetrative sexual assault, and 14.0% for engaging in nonpenetrative sexual offense. Higher rates were observed in men than in women (threat of sexual assault: 22.4% versus 15.4%, penetrative sexual assault: 8.4% versus 6.8%, and nonpenetrative sexual offense: 16.1% versus 13.0%, respectively). Please note that sex difference in penetrative and nonpenetrative sexual offense was not statistically significant. Consistent with studies conducted in Western societies [e.g., (8, 68)], men were found to engage in more sexual offending perpetration than women. In general, men reported to possess significantly more general and subtypes of paraphilic interests (namely voyeurism, exhibitionism, scatologia, fetishism, frotteurism, sadism, biastophilia, urophilia, scatophilia, hebephilia, pedophilia, and zoophilia) than women, while women were found to have more interest in transvestic fetishism than men. The findings that men were reported to have a higher tendency of paraphilic interests than women are in line with findings found in some Western studies [e.g., (69, 70)].

Some findings regarding the influence of psychosocial and sexual health correlates on the possibility of engaging in different types of sexual offending behaviors warrant further discussion, as these provide support for explaining sexual offending behavior. In line with the theoretical proposition (18), low self-control was found to be significantly associated with the tendency to engage in all types of sexual offending behaviors (i.e., threat of sexual assault, penetrative sexual assault, and nonpenetrative sexual offense). This psychosocial risk factor has long been recognized in studies across cultures, including in Asian populations, as a universal predictor of deviant and offending behavior [e.g., (71, 72)]. Specifically on sexual offending, the study by Ha and Beauregard (24) on 69 repeat Canadian sex offenders found that low self-control was a significant predictor of sexual offending behavior, and that sexual offenders low in self-control were impulsive, risky, insensitive, short-sighted, physical, and aggressive during their offending process.

In addition, the participants’ risky sexual behavior was positively associated with all types of sexual offending behaviors. More specifically, penetrative risky sexual behavior was significantly correlated with the participants’ tendency to issue threats of sexual assault and engage in sexual penetration against nonconsenting individuals, whereas nonpenetrative risky sexual behavior was a significant risk factor of nonpenetrative sexual offense. Put differently, those who had engaged in penetrative risky sexual behaviors (e.g., unprotective sex, sex with multiple partners) had a higher likelihood of having issued a threat of sexual assault and of having committing forced sexual penetration; those who had engaged in nonpenetrative risky sexual behaviors (e.g., sexually permissive attitudes, “hooking up” with strangers) were more likely to have committed nonpenetrative sexual offense (e.g., sexual molestation). These findings are consistent with the literature where sexual risk-taking behavior is positively associated with subsequent sexual offending (39, 40, 73). Indeed, many studies have considered deviant fantasies and risky sexual behaviors as precursors to sexual offending (74, 75).

Interestingly, the participants’ general paraphilic interests were only significantly associated with their tendency to commit forced sexual penetration. Perhaps the participants in this sample were more interested in penetrative nature of paraphilic interests (e.g., sadism, biastophilia, pedophilia, and zoophilia). In regard to specific paraphilic interests, the participants’ interest in engaging in zoophilic activities was positively associated with their probability of having engaged in all types of sexual offending behaviors, namely issuing threats of sexual assault, penetrative sexual assault, and nonpenetrative sexual offense. However, the participants who reported more repulsion against urophilic activities were more likely to have issued a threat of sexual assault and to have engaged in penetrative sexual assault, and similar relationships were found for those who reported more repulsion against frotteuristic activities with respect to forced sexual penetration and low biastophilic interest with respect to nonpenetrative sexual offense. The sexual offending literature has reported consistent findings of paraphilic interests and diagnoses of paraphilia among sex offenders. Carvalho (55) postulated that paraphilic interests can subsequently develop into a driving force for some sexual offenses. These interests, and possibly activities, may range from a sexual preference for children to sadism or coercive sex. Notably, high prevalence rates of paraphilias (58 to 98%) have been found among those who committed sexual offenses (76, 77). Relative to previous studies conducted in the West [e.g., (78, 79)], the participants in this study generally reported a lower level of paraphilic interests. Cultural and societal norms might be influential in recognizing and accepting behavior as normal or deviant. In general, Asian cultures (e.g., Chinese culture) adopt a more restrictive view of sexual issues than Western cultures (61). This might be especially relevant for women, who are expected to live up to the gender role assigned to them in a sexually conservative culture. Hence, it is reasonable to posit that revealing one’s paraphilic interests may be culturally sensitive.

Regarding the demographic characteristics, the findings indicated that men were more likely to have issued a threat of sexual assault than women. This is consistent with findings in the literature that the vast majority of sexual offenses are committed by men, with women accounting for a relatively small proportion of those arrested for rape and other sex offenses (4, 80). Similarly, threats of sexual assault are most commonly issued by men against women (81, 82). Interestingly, the participants who reported being non-single (i.e., in a relationship) were found to have a higher tendency than their single counterparts to have engaged in all types of sexual assault (i.e., the threat of sexual assault, and penetrative and nonpenetrative sexual assault). Studies have indicated that most nonhomicidal sex offenders (e.g., rapists and incest offenders) were involved in a sexual relationship at the time of their offense, with the proportion ranging from 73 to 88% [e.g., (83–85)]. Specifically on juvenile sex offenders, Siria et al. (86) found in their sample of 73 Spanish juvenile sex offenders that 30.14% had an intimate partner when they committed the sexual offense, 91.78% had an intimate partner before the sexual offense, and 76.71% had consensual sexual intercourse before the sexual offense. Similar to adult sex offenders, it should be noted that juvenile sex offenders are a heterogenous population with different demographic characteristics depending on the nature of their offense (e.g., penetrative or nonpenetrative sexual assault, or merely issuing a sexual threat).

### Limitations and future research directions

There are several limitations in this study. First, this was a cross-sectional study; and thus, the findings should only be interpreted in correlational terms. Future research should consider adopting a longitudinal design to better comprehend overall causal dynamics of risk factors on different types of sexual offending behaviors. Next, this study was limited to self-reported data wherein the participants’ truthfulness in reporting their sexual interests (e.g., paraphilic interests), practices (e.g., risky sexual behaviors), and offending behaviors (e.g., threat of sexual assault, and nonpenetrative and penetrative sexual assault) can be affected by the research design. Moreover, the use of self-reported data did not allow for a more in-depth investigation of other offense-related factors, such as victim characteristics (e.g., the victim–offender relationship) and other comprehensive offender (e.g., motivation, diagnoses of any mental disorders) and offense characteristics (e.g., situational influences). Thus, future studies could include a response bias measure to reduce participants’ potential reporting biases, use culture-specific measures, and obtain comprehensive responses on other offender characteristics through in-depth follow-up interviews with offenders (e.g., on the nature and severity of sexual offending behavior). Furthermore, this study did not measure the participants’ potential Chinese cultural influences on sexual offending behavior and their sexual health knowledge. The literature has found that potentially adverse cultural circumstance and the individual’s sexual health knowledge can play an influential role in one’s sexually abusive behavior [e.g., (87, 88)]. Therefore, future research shall consider incorporating measures on cultural influences and sexual health knowledge of the participants. Finally, the sample for this study was recruited only from universities and with an unequal sample of women (67%) and men (33%); and thus, the findings cannot be generalized to the wider Hong Kong youth. Future research should consider recruiting a larger and more diverse sample of this population, with an equal sample from both sexes.

### Implications of the findings

This study offers some insights into the general patterns of self-reported sexual offending behaviors among young people in Hong Kong. The findings of this study have important implications for practice. First, it is important to note that appropriate parental supervision and effective parent–child communication are essential to encourage secure and prosocial psychosocial functioning in young people. Research has consistently reported that parental involvement has a significant effect on the subsequent involvement of young people in criminal activities, both as offenders and as victims (89). Hoeve et al. (90) found that the combined effect of parental bonding and control (i.e., supervision, rule-setting, and discipline) can be much stronger than secure parent–child attachment alone in influencing behavior. Hence, a prosocial parenting approach that is responsive to and supportive of children and adolescents is key to strengthening their development of self-control. Training in personal development skills to promote prosocial functioning (e.g., delaying gratification, resiliency) can potentially enhance the self-control of young people. For parents who find it challenging to communicate effectively with their children, social service professionals can act as an effective bridging mechanism between parents and children (91). A higher level of self-control is perceived to be a key protective factor against deviant sexual interests and behaviors [e.g., (24, 25)].

To enhance the understanding among young people of the negative consequences of paraphilic interests (e.g., a sexual preference for children, nonconsenting coercive sex) and risky sexual behaviors (e.g., unprotected sex, many sexual partners), including the possibility of escalating to the actual perpetration of sexual offenses, public awareness of STIs (e.g., HIV) and healthy living (e.g., sexual health) should be enhanced and young people should be educated on these topics much earlier in life. In Hong Kong, sex education has long been criticized for not being sufficiently comprehensive (92). Thus, prevention campaigns should incorporate information about potential risks associated with the early onset of sexual activity and the consumption of alcohol and drugs (93). The need for behavioral changes, such as changing attitudes about safer and more socially acceptable sexual practices (e.g., nonparaphilic activities) and negotiating condom use for protected sex, can be key messages for the younger population. Issues related to hypersexuality and paraphilic interests (e.g., general sexual excitation and inhibition) should also be addressed to better educate young people about other important features of sexuality (50). Given that Chinese culture is traditionally conservative regarding sexual attitudes and practices, and sex is still considered a cultural taboo in most traditional Asian societies, methods to deliver sex education in Hong Kong and other Chinese societies (e.g., mainland China, Macau, Taiwan, Singapore) should be carefully planned with cultural sensitivity in mind.

Sexual sensation-seeking, as manifested in hypersexuality or sexual addiction (e.g., risky sexual behavior, paraphilic interests), has often been regarded as a precursor to sexual offending (94, 95). Kingston et al. (96) posited that the positive consequences of relational sexual activity with appropriate and consenting partners (e.g., increased sense of intimacy, minimized loneliness) can be considered as protective factors against future sexual offending; and thus, can be incorporated as treatment targets in sexual offender programs. For sexually high-risk individuals, inaccurate perceptions about deviant sexual interests and behaviors should be addressed [(49); Marten et al., 2006]. These social norm interventions may have significant remedial outcomes through diminishing the frequency of those who already possess paraphilic interests or engage in a risky sexual behavior, and preventive outcomes by correcting misperceptions of those who do not yet regularly engage in such behavior. In addition, other preventive measures, such as public mental health seminars, can be organized to highlight that relational sexual activity with appropriate and consenting partners can be a very positive experience with positive outcomes. Assertiveness training that focuses on relationships with the opposite sex or with sexual partners can be useful to foster greater sexual assertiveness as a form of self-protection in relationships. Nonetheless, in designing these and similar interventions, mental health professionals should expect that Western practices may not be completely applicable in the Asian context.

## Data availability statement

The raw data supporting the conclusions of this article will be made available by the authors, without undue reservation.

## Ethics statement

The studies involving human participants were reviewed and approved by Human Subjects Ethics Sub-Committee, City University of Hong Kong. Written informed consent to participate in this study was provided by the participants’ legal guardian/next of kin.

## Author contributions

The author confirms being the sole author of this work and has approved it for publication.

## Funding

This research was supported by City University of Hong Kong, Hong Kong, SAR [7004958(SS)] with funding provided to HC.

## Conflict of interest

The author declares that the research was conducted in the absence of any commercial or financial relationships that could be construed as a potential conflict of interest.

## Publisher’s note

All claims expressed in this article are solely those of the authors and do not necessarily represent those of their affiliated organizations, or those of the publisher, the editors and the reviewers. Any product that may be evaluated in this article, or claim that may be made by its manufacturer, is not guaranteed or endorsed by the publisher.

## Footnotes

^1^According to Pye [62], traditional Chinese culture consists of different competing schools of thought, including Confucianism, Taoism, and Buddhism. Confucianism. These schools of thought generally forms the backbone of Chinese cultural tradition that emphasizes on human relationships, social structures, virtuous behavior, and work ethic. In simple, relationships are structured to ensure a harmonious society that focuses the importance of filial piety and loyalty.

^2^Penetrative risky sexual behavior simply refers to risky sexual behavior involving penetration (e.g., vaginal sex), and nonpenetrative risky sexual behavior refers to risky sexual behavior that does not involve sexual penetration (e.g., breast fondling). General risky sexual behavior is overall risky sexual behavior, including both the penetrative and nonpenetrative subtypes.

^3^Fourteen paraphilic interests were examined: voyeurism, exhibitionism, scatologia, fetishism, transvestic fetishism, frotteurism, sadism, masochism, biastophilia, urophilia, scatophilia, hebephilia, pedophilia, and zoophilia. General paraphilic interest is the overall interest in paraphilic activities.

^4^The United Nations [63] defines “youth” as individuals between the ages of 15 and 24.

^5^The age distribution of the participants was: 17 years (2.5%, *n* = 22), 18 years (26.8%, *n* = 231), 19 years (27.8%, *n* = 240), and 20 years (42.9%, *n* = 370).

^6^These subtypes were (a) voyeurism (sexual arousal involving the observation of an unsuspecting individual who is naked, undressing, or engaging in sexual activity; one item), (b) exhibitionism (sexual arousal involving the exposure of one’s genitals to an unsuspecting individual; one item), (c) scatologia (sexual arousal involving the making of unsolicited and obscene telephone calls; one item), (d) fetishism (sexual arousal involving nonliving objects such as shoes and undergarments; three items), (e) transvestic fetishism (sexual arousal involving cross-dressing activities; two items), (f) frotteurism (sexual arousal involving activities of touching and rubbing against an unsuspecting individual; one item), (g) sadism (sexual arousal involving activities of inflicting harm and humiliation on another individual; six items), (h) masochism (sexual arousal involving activities of being humiliated, beaten, bound, or otherwise made to suffer; six items), (i) biastophilia (sexual arousal involving having sexual intercourse with a nonconsenting individual; two items), (j) urophilia (sexual arousal involving contact with urine; two items), (k) scatophilia (sexual arousal involving contact with feces; two items), (l) hebephilia (sexual arousal involving having sexual intercourse with pubescent children; two items), (m) pedophilia (sexual arousal involving having sexual intercourse with prepubescent children; two items), and (n) zoophilia (sexual arousal involving having sexual intercourse with animals; one item).

## References

[1] Chan HCO. Sexual offending in Asia: A psycho-criminological perspective. West Sussex, UK: John Wiley & Sons (2023).

[2] Chan HCO. The victim-offender overlap in sexual offending: exploring a community-based sample of young adults in Hong Kong. Sex Abus. (2021) 33:923–9. doi: 10.1177/1079063220981889, PMID: 33353485

[3] Chan KL. Association between childhood sexual abuse and adult sexual victimization in a representative sample in Hong Kong Chinese. Child Abuse Negl. (2011) 35:220–9. doi: 10.1016/j.chiabu.2010.11.005, PMID: 21481928

[4] Cortoni F Hanson RK A review of the recidivism rates of adult female sexual offenders (research rep. No. R-169). Correctional Service Canada: Ottawa Available at: https://www.csc-scc.gc.ca/research/092/r169_e.pdf (accessed on 8 March 2022).

[5] Lau Y Chan KS. Influence of intimate partner violence during pregnancy and early postpartum depressive symptoms on breastfeeding among Chinese women in Hong Kong. J Midwifery Womens Health. (2007) 52:e15–20. doi: 10.1016/j.jmwh.2006.09.001, PMID: 17336812

[6] Tang CS. Childhood experience of sexual abuse among Hong Kong Chinese college students. Child Abuse Negl. (2002) 26:23–37. doi: 10.1016/S0145-2134(01)00306-4, PMID: 11860160

[7] Chan KL Yan E Brownridge DA Tiwari A Fong DYT. Childhood sexual abuse associated with dating partner violence and suicidal ideation in a representative household sample in Hong Kong. J Interpers Violence. (2011) 26:1763–84. doi: 10.1177/0886260510372943, PMID: 20587453

[8] Finkelhor D Ormrod R Chaffin M. Juveniles who commit sex offenses against minors. Juvenile Justice Bulletin: Office of Juvenile Justice and Delinquency Prevention. US Department of Justice (2009).

[9] Federal Bureau of Investigation. Crime in the United States, 2013. Washington, DC: U.S. Government Printing Office (2014).

[10] Barbaree HE Hudson SM Seto MC. Sexual assault in society: the role of the juvenile offender In: Barbaree HE Marshall WL Hudson SM, editors. The juvenile sex offender. New York: Guilford (1993). 1–24.

[11] Piquero A Farrington DP Jennings W Diamond B Craig J. Sex offenders and sex offending in the Cambridge study in delinquent development: prevalence, frequency, (dis)continuity over the life-course. J Crime Justice. (2012) 35:412–6. doi: 10.1080/0735648X.2012.688527

[12] Hong Kong Police Force. (2022). Hong Kong crime statistics. Available at: https://www.police.gov.hk/ppp_en/09_statistics/index.html

[13] Carpentier J Proulx J. Correlates and recidivism among adolescents who have sexually offended. Sex Abus J Res Treat. 23:434–5. doi: 10.1177/1079063211409950, PMID: 21960517

[14] Boakye KE. Juvenile sexual offending in Ghana: prevalence, risks and correlates. Child Abuse Negl. (2020) 101:104318. doi: 10.1016/j.chiabu.2019.104318, PMID: 31887654

[15] Riser DK Pegram SE Farley JP. Adolescent and young adult male sex offenders: understanding the role of recidivism. J Child Sex Abus. (2013) 22:9–31. doi: 10.1080/10538712.2013.735355, PMID: 23350537

[16] Ryan EP Otonichar JM. Juvenile sex offenders. Curr Psychiatry Rep. (2016) 18:67. doi: 10.1007/s11920-016-0706-1 27222141

[17] Tharp AT Valle LA Brookmayer KA Massetti GM Matjasko JL. A systematic qualitative review of risk and protective factors for sexual violence perpetration. Trauma Violence Abuse. (2013) 14:133–7. doi: 10.1177/1524838012470031, PMID: 23275472

[18] Gottfredson MR Hirschi T. A general theory of crime. Stanford, CA: Stanford University Press (1990).

[19] Muraven M Pogarsky G Shmueli D. Self-control depletion and the general theory of crime. J Quant Criminol. (2006) 22:263–7. doi: 10.1007/s10940-006-9011-1

[20] Vazsonyi AT Klanjsek R. A test of self-control theory across different socioeconomic strata. Justice Q. (2008) 25:101–1. doi: 10.1080/07418820801954571

[21] Chan HCO. Risky sexual behavior of young adults in Hong Kong: an exploratory study of psychosocial risk factors. Front Psychol. (2021b) 12:658179. doi: 10.3389/fpsyg.2021.658179, PMID: 33828516 PMC8019819

[22] Chiesi F Bonacchi A Lau C Tosti AE Marra F Saklofske DH. Measuring self-control across gender, age, language, and clinical status: a validation study of the Italian version of the brief self-control scale (BSCS). PLoS One. (2020) 15:e0237729. doi: 10.1371/journal.pone.0237729, PMID: 32822379 PMC7446922

[23] de Ridder DTD Lensvelt-Mulders G Finkenauer C Stok FM Baumeister RF. Taking stock of self-control: a meta-analysis of how trait self-control relates to a wide range of behaviors. Personal Soc Psychol Rev. (2012) 16:76–99. doi: 10.1177/1088868311418749 21878607

[24] Ha OK Beauregard E. Sex offending and low self-control: an extension and test of the general theory of crime. J Crim Just. (2016) 47:62–73. doi: 10.1016/j.jcrimjus.2016.07.005

[25] Healey J Beauregard E. Impulsivity as an etiological factor in sexual homicide. J Crim Just. (2016) 48:30–6. doi: 10.1016/j.jcrimjus.2016.12.002

[26] DeLisi M Wright J. Social control theory of sexual homicide offending In: Bruinsma G Weisburd D, editors. Encyclopedia of criminology and criminal justice. New York: Springer (2014)

[27] Abajobir AA Kisely S Maravilla JC Williams G Najman JM. Gender differences in the association between childhood sexual abuse and risky sexual behaviours: a systematic review and meta-analysis. Child Abuse Negl. (2017) 63:249–13. doi: 10.1016/j.chiabu.2016.11.023, PMID: 27908449

[28] Hoyle RH Fejfar MC Miller JD. Personality and sexual risk taking: a quantitative review. J Pers. (2000) 68:1203–31. doi: 10.1111/1467-6494.00132 11130738

[29] Smith EA Udry JR Morris NM. Pubertal development and friends: a biosocial explanation of adolescent sexual behavior. J Health Soc Behav. (1985) 26:183–2. doi: 10.2307/2136751, PMID: 4067236

[30] Romer D. Adolescent risk taking, impulsivity, and brain development: implications for prevention. Dev Psychobiol. (2010) 52:276. doi: 10.1002/dev.20442, PMID: 20175097 PMC3445337

[31] Steinberg L. A social neuroscience perspective on adolescent risk-taking. Dev Rev. (2008) 28:78–6. doi: 10.1016/j.dr.2007.08.002, PMID: 18509515 PMC2396566

[32] Smith GT Anderson KG. Adolescent risk for alcohol problems as acquired preparedness: a model and suggestions for intervention In: Monty PM Colby SM O’Leary TA, editors. Adolescents, alcohol, and substance abuse: Reaching teens through brief interventions. New York, NY: Guilford Press (2001)

[33] Dahl RE. Adolescent brain development: a period of vulnerabilities and opportunities. Keynote address. Ann N Y Acad Sci. (2004) 1021:1–22. doi: 10.1196/annals.1308.001, PMID: 15251869

[34] Smetana JG Campione-Barr N Metzger A. Adolescent development in interpersonal and societal contexts. Annu Rev Psychol. (2006) 57:255–4. doi: 10.1146/annurev.psych.57.102904.190124, PMID: 16318596

[35] DiClemente R Milhausen RR Salazar LF Spitalnick J Sales JM Crosby RA. Development of the sexual sensation-seeking scale for African American adolescent women. Int J Sex Health. (2010) 22:248–1. doi: 10.1080/19317611.2010.491388

[36] Fetene N Mekonnen W. The prevalence of risky sexual behaviors among youth center reproductive health clinics users and non-users in Addis Ababa, Ethiopia: a comparative cross-sectional study. PLoS One. (2018) 13:e0198657. doi: 10.1371/journal.pone.0198657, PMID: 29879164 PMC5991709

[37] Martinez G Copen CE Abma JC Teenagers in the United States: sexual activity, contraceptive use, and childbearing, 2006–2010 National Survey of family growth. Vital and health statistics (series 23, number 31). Available at: https://www.cdc.gov/nchs/data/series/sr_23/sr23_031.pdf (2011) (accessed 14 May 2020). 22256688

[38] Pinyopornpanish K Thanamee S Jiraporncharoen W Thaikla K McDonald J Aramrattana A. Sexual health, risky sexual behavior and condom use among adolescents, young adults, and older adults in Chiang Mai, Thailand: findings from a population based survey. BMC Res Notes. (2017) 10:682. doi: 10.1186/s13104-017-3055-1, PMID: 29202883 PMC5715516

[39] Smallbone S Cale J. An integrated life-course developmental theory of sexual offending In: Blockland A Lussier P, editors. Sex offenders: a criminal career approach. West Sussex, UK: John Wiley & Sons (2015)

[40] Lussier P Cale J. Beyond sexual recidivism: a review of the sexual criminal career parameters of adult sex offenders. Aggress Violent Behav. (2013) 18:445–7. doi: 10.1016/j.avb.2013.06.005

[41] Malamuth NM Linz D Heavey CL Barnes G Acker M. Using the confluence model of sexual aggression to predict men’s conflict with women: a 10-year follow-up study. J Pers Soc Psychol. (1995) 69:353–9. doi: 10.1037/0022-3514.69.2.353, PMID: 7643309

[42] Davis KC Neilson EC Wegner R Danube CL. The intersection of men’s sexual violence perpetration and sexual risk behavior: a literature review. Aggress Violent Behav. (2018) 40:83–90. doi: 10.1016/j.avb.2018.04.001, PMID: 30713462 PMC6350826

[43] Bongers IL Koot HM Van der Ende J Verhulst FC. The normative development of child and adolescent problem behavior. J Abnorm Psychol. (2003) 112:179–2. doi: 10.1037/0021-843X.112.2.179 12784827

[44] Kotchick BA Shaffer A Miller KS Forehand R. Adolescent sexual risk behavior: a multi-system perspective. Clin Psychol Rev. (2001) 21:493–9. doi: 10.1016/S0272-7358(99)00070-7, PMID: 11413865

[45] Murphy D Rotheram-Borus MJ Reid H. Adolescent gender differences in HIV-related sexual risk acts, social-cognitive factors and behavioral skills. J Adolesc. (1998) 21:197–208. doi: 10.1006/jado.1997.0141, PMID: 9585496

[46] Crockett LJ Raffaelli M Shen Y. Linking self-regulation and risk proneness to risky sexual behavior: pathways through peer pressure and early substance use. J Res Adolesc. (2006) 16:503–5. doi: 10.1111/j.1532-7795.2006.00505.x

[47] American Psychiatric Association. Diagnostic and statistical manual of mental disorders. 5th ed. Washington, DC: American Psychiatric Publishing (2013).

[48] Bouchard KN Dawson SJ Lalumière ML. The effects of sex drive and paraphilic interests on paraphilic behaviours in a nonclinical sample of men and women. Can J Hum Sex. (2017) 26:97–1. doi: 10.3138/cjhs.262-a8

[49] Chan HCO. Paraphilic interests: the role of psychosocial factors in a sample of young adults in Hong Kong. Sex Res Soc Policy. 19:159–8. doi: 10.1007/s13178-020-00532-z

[50] Dawson SJ Bannerman BA Lalumière ML. Paraphilic interests: an examination of sex differences in a nonclinical sample. Sex Abuse J Res Treat. (2016) 28:20–45. doi: 10.1177/1079063214525645 24633420

[51] Levaque E Dawson SJ Wan C Lalumière ML. Sex drive as a possible mediator of the gender difference in the prevalence of paraphilic interests in a nonclinical sample. Arch Sex Behav. (2021) 51:867–7. doi: 10.1007/s10508-021-02074-w 34750773

[52] Bártová K Androvičová R Krejčová L Weiss P Klapilová K. The prevalence of paraphilic interests in the Czech population: preference, arousal the use of pornography, fantasy, and behavior. J Sex Res. (2021) 58:86–96. doi: 10.1080/00224499.2019.1707468, PMID: 31916860

[53] Joyal CC Carpentier J. Concordance and discordance between paraphilic interests and behaviors: a follow-up study. J Sex Res. (2021). Advance online publication) 59:385–13. doi: 10.1080/00224499.2021.1986801, PMID: 34637647

[54] Seto MC Curry S Dawson SJ Bradford JM Chivers ML. Concordance of paraphilic interests and behaviors. J Sex Res. (2021) 58:424–7. doi: 10.1080/00224499.2020.1830018, PMID: 33112690

[55] Carvalho J. Paraphilic sexual interests and sexual offending: implications for risk assessment and treatment. J Sex Med. (2018) 15:927–8. doi: 10.1016/j.jsxm.2018.02.002, PMID: 29861355

[56] Drury A Heinrichs T Elbert M Tahja K DeLisi M Caropreso D. Adverse childhood experiences, paraphilias, and serious criminal violence among federal sex offenders. J Crim Psychol. (2017) 7:105–9. doi: 10.1108/JCP-11-2016-0039

[57] Cantor JM McPhail IV. Non-offending pedophiles. Curr Sex Health Rep. 8:121–8. doi: 10.1007/s11930-016-0076-z

[58] Seto MC. Internet sex offenders. Washington, DC: American Psychological Association (2013).

[59] Reiss IL. Journey into sexuality: An exploratory voyage. Englewood Cliffs, NJ: Prentice Hill (1986).

[60] Baazeem A. Challenges to practicing sexual medicine in the Middle East. Sex Med Rev. (2016) 4:221–8. doi: 10.1016/j.sxmr.2016.04.001, PMID: 27871955

[61] Ho CC Singam P Hong GE Zainuddin ZM. Male sexual dysfunction in Asia. Asian J Androl. (2011) 13:537–2. doi: 10.1038/aja.2010.135 21643001 PMC3739628

[62] Pye LW. China: An introduction. Boston, MA: Little Brown (1972).

[63] United Nations. (2021). Global issues: Youth. available at: https://www.un.org/en/global-issues/youth (accessed 2 March 2022).

[64] Grasmick HG Tittle CR Bursik RJ Arneklev BJ. Testing the core implications of Gottfredson and Hirschi’s general theory of crime. J Res Crime Delinq. (1993) 30:5–29. doi: 10.1177/0022427893030001002

[65] Turchik JA Garske JP. Measurement of sexual risk taking among college students. Arch Sex Behav. (2009) 38:936–8. doi: 10.1007/s10508-008-9388-z 18563548

[66] Seto MC Lalumière ML Harris GT Chivers ML. The sexual responses of sexual sadists. J Abnorm Psychol. (2012) 121:739–3. doi: 10.1037/a0028714, PMID: 22708887

[67] Gravetter FJ Wallnau LB. Essentials of statistics for the behavioral sciences. 10th ed. Belmont, CA: Wadsworth, Cengage Learning (1993).

[68] Fox B. What makes a difference? Evaluating the key distinctions and predictors of sexual and non-sexual offending among male and female juvenile offenders. J Crim Psychol. (2017) 7:134–13. doi: 10.1108/JCP-12-2016-0047

[69] Joyal CC Carpentier J. The prevalence of paraphilic interests and behaviors in the general population: a provincial survey. J Sex Res. (2017) 54:161–1. doi: 10.1080/00224499.2016.1139034, PMID: 26941021

[70] Richters J Grulich AE Visser RO Smith A Rissel CE. Sex in Australia: autoerotic, esoteric, and other sexual practices engaged in by a representative sample of adults. Aust N Z J Public Health. (2007) 27:180–13. doi: 10.1111/j.1467-842X.2003.tb00806.x, PMID: 14696709

[71] Chan HCO. Exploring the overlap between victimization and offending among Hong Kong adolescents. J Crim Just. (2019a) 61:72–80. doi: 10.1016/j.jcrimjuc.2019.03.003

[72] Sacarellos CD Wright JP Almosaed NF Moghrabi SS Bashatah FS Morgan MA. Crime in the kingdom: the effects of low self-control in a Saudi Arabian sample of youth. Youth Violence Juvenile Justice. (2016) 14:291–2. doi: 10.1177/1541204015616663

[73] Chan HCO. A global casebook of sexual homicide. Singapore: Springer Nature (2019b).

[74] Abel GG Blanchard EB. The role of fantasy in the treatment of sexual deviation. Arch Gen Psychiatry. (1974) 30:467–5. doi: 10.1001/archpsyc.1974.01760100035007 4815552

[75] Marshall WL Barbaree HE Eccles A. Early onset and deviant sexuality in child molesters. J Interpers Violence. (1991) 6:323–5. doi: 10.1177/088626091006003005

[76] Jackson RL Richards HJ. Diagnostic and risk profiles among civilly committed sex offenders in Washington state. Int J Offender Ther Comp Criminol. (2007) 51:313–3. doi: 10.1177/0306624X06292874, PMID: 17478861

[77] McElroy SL Soutullo C Taylor P Nelson EB Beckman DA Brunsman LA. Psychiatric features of 36 men convicted of sexual offenses. J Clin Psychiatry. (1999) 60:414–13. doi: 10.4088/JCP.v60n0613, PMID: 10401925

[78] Kafka MP. Hypersexual disorder: a proposed diagnosis for DSM-V. Arch Sex Behav. (2009) 39:377–13. doi: 10.1007/s10508-009-9574-7 19937105

[79] Långström N Hanson KR. High rates of sexual behavior in the general population: correlates and predictors. Arch Sex Behav. (2006) 35:37–52. doi: 10.1007/s10508-006-8993-y, PMID: 16502152

[80] Frey LL. The juvenile female sexual offender: characteristics, treatment and research In: Gannon T Cortoni F, editors. Female sexual offenders: Theory, assessment and treatment. New York: Wiley Blackwell (2010)

[81] Nurius PS Norris J Young DS Graham TL Gaylord J. Interpreting and defensively responding to threat: examining appraisals and coping with acquaintance sexual aggression. Violence Vict. (2000) 15:187–8. doi: 10.1891/0886-6708.15.2.187, PMID: 11108501

[82] VanZile-Tamsen C Testa M Livingston JA. The impact of sexual assault history and relationship context on appraisal of and responses to acquaintance sexual assault risk. J Interpers Violence. (2005) 20:813–2. doi: 10.1177/0886260505276071, PMID: 15914703

[83] Firestone P Bradford JM Greenberg DM Larose MR. Homicidal sex offenders: psychological, phallometric, and diagnostic features. J Am Acad Psych Law. (1998) 6:264–2. doi: 10.1016/S1353-1131(99)90030-5 9894211

[84] Oliver CJ Beech AR Fishers D Beckett R. A comparison of rapists and sexual murderers on demographic and selected psychometric measures. Int J Offender Ther Comp Criminol. 51:298–2. doi: 10.1177/0306624X06289157, PMID: 17478860

[85] Vettor S Beech AR Woodhams J. Rapists and sexual murderers: combined pathways to offending In: Proulx J Beauregard E Lussier P Leclerc B, editors. Pathways to sexual aggression. Oxon, UK: Routledge (2014)

[86] Siria S Echeburú E Amor PJ. Characteristics and risk factors in juvenile sexual offenders. Psicothema. (2020) 32:314–1. doi: 10.7334/psicothema2019.349 32711665

[87] Lunsky Y Frijters J Griffiths DM Watson SL Williston S. Sexual knowledge and attitudes of men with intellectual disability who sexually offend. J Intellect Develop Disabil. (2007) 32:74–81. doi: 10.1080/13668250701408004, PMID: 17613678

[88] Ward T Beech AR. The integrated theory of sexual offending-revised In: Boer DP, editor. The Wiley handbook on the theories, assessment and treatment of sexual offending. New York: Wiley-Blackwell (2016).

[89] Craig JM. Which bond matters more? Assessing the differential strengths of parental bonding measures on adolescent delinquency over time. Youth Violence Juvenile Justice. (2015) 14:225–2. doi: 10.1177/1541204014565670

[90] Hoeve M Stams GJM van der Put CE Dubas JS van der Laan PH Gerris JRM. A meta-analysis of attachment to parents and delinquency. J Abnorm Child Psychol. (2012) 40:771–5. doi: 10.1007/s10802-011-9608-1, PMID: 22278802 PMC3375078

[91] Chan HCO Chui WH. Psychological correlates of violent and nonviolent Hong Kong juvenile probationers. Behav Sci Law. (2012) 30:103–13. doi: 10.1002/bsl.2003, PMID: 22362616

[92] Andres EB Choi EPH Fung AWC Lau KWC Ng NHT Yeung M. Comprehensive sexuality education in Hong Kong: study protocol for process and outcome evaluation. BMC Public Health. (2021) 21. doi: 10.1186/s12889-021-10253-6, PMID: 33482802 PMC7820515

[93] Staton M Leukefeld C Logan T Zimmerman R Lynam D Milich R. Risky sex behaviour and substance use among young adults. Health Soc Work. (1999) 24:147–4. doi: 10.1093/hsw/24.2.147 10340165

[94] Kingston DA Bradford JM. Hypersexuality and recidivism among sexual offenders. Sex Addict. Compul J Treat Prevent. (2013) 20:91–5. doi: 10.1080/10720162.2013.768131

[95] Marshall LE Marshall WL. Sexual addiction in incarcerated sexual offenders. SexAddict Compul J Treat Prevent. (2007) 13:377–13. doi: 10.1080/10720160601011281

[96] Kingston DA Yates PM Firestone P. The self-regulation model of sexual offender treatment: relationship to risk and need. Law Hum Behav. (2012) 36:215–24. doi: 10.1037/h0093960, PMID: 22667811

